# The Difficult Evolution of Intensive Cardiac Care Units: An Overview of the BLITZ-3 Registry and Other Italian Surveys

**DOI:** 10.1155/2017/6025470

**Published:** 2017-11-20

**Authors:** Gianni Casella, Silvia Zagnoni, Giuseppe Fradella, Giampaolo Scorcu, Alessandra Chinaglia, Pier Camillo Pavesi, Giuseppe Di Pasquale, Luigi Oltrona Visconti

**Affiliations:** ^1^Cardiology Department, Maggiore Hospital, Bologna, Italy; ^2^Cardiology Department 1, Careggi Hospital, Firenze, Italy; ^3^Cardiology Department, G. Brotzu Hospital, Cagliari, Italy; ^4^Cardiology Department, Maria Vittoria Hospital, Torino, Italy; ^5^Cardiology Department, Fondazione IRCCS Policlinico San Matteo, Pavia, Italy

## Abstract

Coronary care units, initially developed to treat acute myocardial infarction, have moved to the care of a broader population of acute cardiac patients and are currently defined as Intensive Cardiac Care Units (ICCUs). However, very limited data are available on such evolution. Since 2008, in Italy, several surveys have been designed to assess ICCUs' activities. The largest and most comprehensive of these, the BLITZ-3 Registry, observed that patients admitted are mainly elderly males and suffer from several comorbidities. Direct admission to ICCUs through the Emergency Medical System was rather rare. Acute coronary syndromes (ACS) account for more than half of the discharge diagnoses. However, numbers of acute heart failure (AHF) admissions are substantial. Interestingly, age, resources availability, and networking have a strong influence on ICCUs' epidemiology and activities. In fact, while patients with ACS concentrate in ICCUs with interventional capabilities, older patients with AHF or non-ACS, non-AHF cardiac diseases prevail in peripheral ICCUs. In conclusion, although ACS is still the core business of ICCUs, aging, comorbidities, increasing numbers of non-ACS, technological improvements, and resources availability have had substantial effects on epidemiology and activities of ICCUs. The Italian surveys confirm these changes and call for a substantial update of ICCUs' organization and competences.

## 1. Introduction

During the sixties, coronary care units (CCUs) have been initially developed to treat arrhythmic complications in patients with acute myocardial infarction (AMI) [1]. Afterwards, the implementation of reperfusion therapy in ST-elevation and early revascularization in non-ST-elevation acute coronary syndromes (ACS) further reduced mortality and morbidity of patients admitted into these units [2]. However, due to the demographical changes of the population, these CCUs began to deal with elderly patients with several comorbidities [3]. In addition, several subjects with acute heart failure (AHF), major arrhythmias, high-risk pulmonary embolism, or other acute cardiac conditions need intensive care as well [4]. Thus, at present, admission to CCUs has extended to a large number of critical acute cardiac diseases that need highly specialized intensive care [2]. Therefore, the definition of CCU has moved to a more comprehensive term, that is, Intensive Cardiac Care Unit (ICCU) [5]. On the other hand, such evolution of acute cardiac care and ICCU has substantial drawbacks [3] and raises a strong heterogeneity of care between or even within countries [6]. These discrepancies due to differences in competences, resources availability, and healthcare organization may cause disparities in acute cardiac care as well [7]. Furthermore, objective data on acute cardiac care and ICCU are so scarce that producing evidence-based guidelines is an issue [8].

Starting from this background, in 2008, the Italian ANMCO (Association of Hospital Cardiologists) called for a national survey, the BLITZ-3 Registry [9], to better understand the epidemiology and patterns of care of patients admitted to ICCUs. This seminal survey promoted several other regional registries in our country. The aim of the current paper is to review these Italian experiences on ICCUs and to discuss their most critical issues.

## 2. The BLITZ-3 Registry Overview

**Figure 1:**
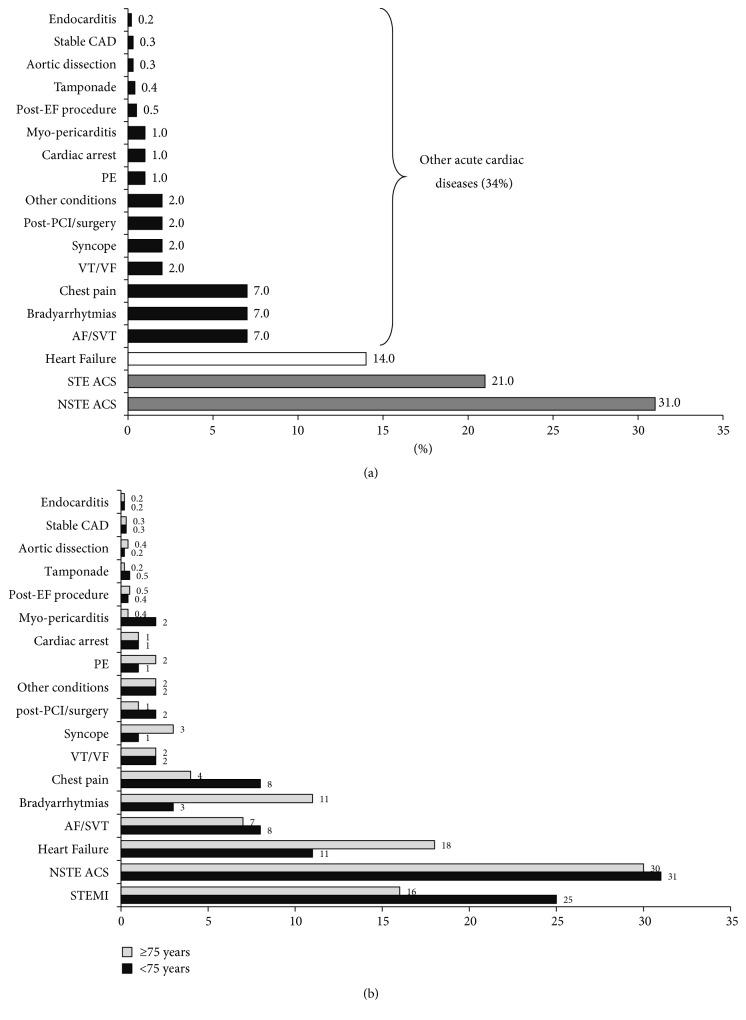
Discharge diagnosis of patients enrolled in the Italian BLITZ-3 Registry. (a) General population. Modified from Casella et al. for the BLITZ-3 investigators [9]. (b) Effects of aging. Modified from Casella et al. for the BLITZ-3 investigators [12]. CAD: coronary artery disease; PCI: percutaneous coronary intervention; VT: ventricular tachycardia; VF: ventricular fibrillation; AF: atrial fibrillation; SVT: supraventricular tachycardia; STE ACS: ST-elevation acute coronary syndrome; NSTE: ACS non-ST-elevation acute coronary syndrome; PE: pulmonary embolism; Post-EF procedure: postelectrophysiological procedure complications.

The BLITZ-3 survey [9] was a multicenter, prospective, observational, nationwide study that enrolled 6986 consecutive patients, admitted to 81% Italian ICCUs during a 2-week interval in 2008. Patients enrolled were mainly elderly males, with several relevant, chronic comorbidities (diabetes by far the most prevalent) (Table 1). ACS, mainly non-ST-elevation ACS, was the most common reason for admission (Figure 1(a)). As expected, patients were most often triaged from the emergency room (ER), while few of them, mainly with ST-elevation ACS, were directly admitted to ICCUs by Emergency Medical Services (EMS). Echocardiography, coronary angiography, and percutaneous coronary intervention (PCI) were the most used procedures. Few patients needed temporary pacing, electrical cardioversion, ventilation, intra-aortic balloon pump, or ultrafiltration. The incidence of ventricular fibrillation or complete atrioventricular block, once the most common complications in ICCU, was rather low. However, new onset or worsening of heart failure, shock, or worsening of renal function was frequently observed. Stroke and sepsis were rather rare.

In the BLITZ-3 survey, ST-elevation ACS (observed at any time interval from onset of symptoms) accounted for 21% of the admissions. As compared to the general population, ST-elevation ACS patients were younger and had a better risk profile, and most of them were still admitted to the ICCU through the ER (Table 1). However, 49% of ST-elevation subjects had a transmitted prehospital ECG. Reperfusion (15% fibrinolysis and 45% primary PCI) was applied in 60% of cases. The in-ICCU crude mortality was 8% for nonreperfused ST-elevation ACS, 3.1% for patients treated with primary PCI, and 3.5% for thrombolysis. Besides, non-ST-elevation ACS were the most frequent cause of admission to the ICCU (31%). These patients were older, had a worse risk profile than their ST-elevation counterparts, and were rarely admitted directly by EMS (Table 1). Interestingly, 50% of them underwent coronary angiography and 32% PCI.

Acute heart failure (AHF), the 2nd rated admission diagnosis after ACS, accounted for 14% of cases. These subjects were the oldest, with the worst risk profile (Table 1). Most of them underwent echocardiography, while only 14% were ventilated. Ten percent of cases were submitted to coronary angiography and 1% to PCI during their ICCU stay. Ultrafiltration and counterpulsation were seldom used. Diuretics and nitrates were used in the majority of cases. In several patients, shock or worsening of heart failure was observed during hospitalization, and 18% of cases had worsening of the renal function. The in-ICCU crude mortality of AHF was 5.4%. Advanced age and elevated creatinine values were associated with a higher risk of in-ICCU death [11]. Other acute non-ACS, non-AHF cardiac diseases accounted for 34% of the admissions. Among these conditions, bradyarrhythmias, supraventricular arrhythmias, and chest pain were the most common diagnosis (Figure 1(a)). Interestingly, this heterogeneous group of patients had an overall risk profile comparable to that of the general population (Table 1).

## 3. Are ACS Still the “Core Business” of Modern ICCUs?

The Italian BLITZ-3 study [9] shows that although ACS were still the most common admission diagnosis, the epidemiology of ICCU is changing, with increasing numbers of elderly, non-ACS subjects with multiple noncardiac comorbidities admitted. Katz et al. were the first to describe these changes reporting historical data from the Duke University Center [3]. Similarly, Valente et al. reviewed the caseload of a tertiary ICCU in Italy and observed that although ACS was still the most common admitting diagnosis, the number of patients with respiratory failure, acute renal dysfunction, or sepsis, or in need for mechanical ventilation or ultrafiltration, has continuously increased [14]. However, the BLITZ-3 survey was the first study that observed this changing epidemiology at a national level. Recently, Roubille et al. [15], reporting the largest experience in ICCUs' activity to date, extended these observations even further. In fact, the authors observed that in France the number of non-ACS admissions is large and the risk profile of the population cared for is definitely high with remarkable numbers of elderly and females. Similar findings have been observed when analyzing a large, tertiary-care, academic ICCU in the United States. In addition, this study reported that acute noncardiovascular illnesses are associated with higher mortality and increased length of hospital stay [16]. Thus, this changing epidemiology of ICCU could challenge the existence of a specific CCU, as we used to know in many cases [17]. First, it is common experience that prevalence of type I AMI (acute myocardial infarction) is falling. Secondly, the effectiveness of mechanical reperfusion in ST-elevation ACS, and of early interventions in non-ST-elevation ACS, shifts these syndromes from peripheral ICCUs to facilities with interventional capabilities [10, 18]. In addition, patients with advanced cardiac disease complicated by severe noncardiovascular comorbidities (e.g., sepsis or kidney injury in a patient with acute or chronic heart failure) are increasingly common. Furthermore, the better results observed in non-ACS critical cardiac patients when they are cared for under cardiological supervision [16] and the increase in AHF patients, or of patients with acute cardiac complications of noncardiac disease (like type 2 AMI), once admitted to regular wards, emphasize the need for high-level acute cardiac care event for these non-ACS populations. Finally, in the near future, acute cardiac care could extend its influence on other acute vascular diseases like stroke or type B aortic dissection. Thus, all these trends highlight the overlapping populations between the contemporary ICCU and traditional medical intensive care unit and call for a strong evolution of the clinical competence of cardiologists working in ICCUs.

**Figure 2:**
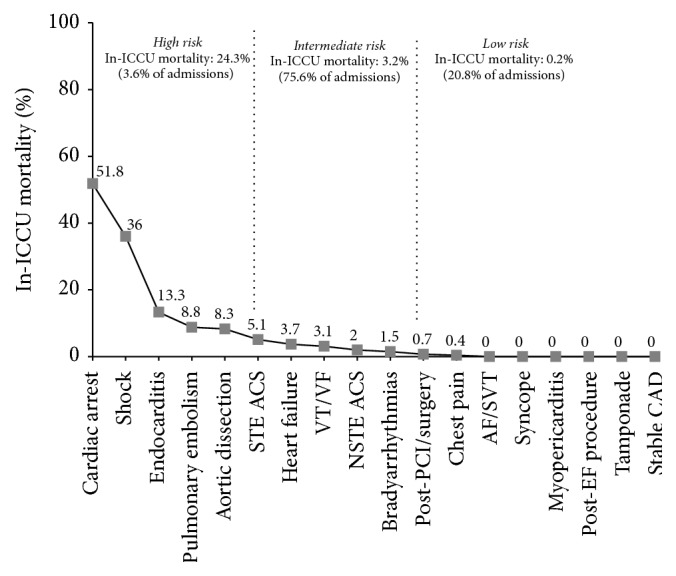
Classes of risk of mortality during admission to the ICCU. Modified from Oltrona Visconti et al. [13]. Legend as Figure 1.

In addition, patients with acute cardiac conditions represent a very heterogeneous group (Figure 2) with different critical subsets that range from emergent, very-high-risk situations like aortic dissection, tamponade, resuscitated cardiac arrest, or arrhythmias storming, to low-risk subsets where patients require only specialized monitoring of their conditions (i.e., high-risk chest pain or after complex interventional procedures). The management of such different cases represents a challenge since several of them could suffer the shortage of beds in ICCUs, whereas others may receive excessive care. Thus, we feel that the current model of care, based exclusively on intensive care units and conventional wards, could be improved with the development of intermediate units. These units could handle many cardiac patients requiring monitoring and an intensity of medical care superior to that available in a regular ward, but without the medical or technical costs of a traditional ICCU [19].

## 4. Effect of Aging on Epidemiology of Admission and ICCU Patterns of Care

Interestingly, all available data on ICCUs underline the notion that the admitted population is aging and this has substantial clinical consequences. In fact, within the BLITZ-3 Registry population, 43% were elderly (≥75 years) and their risk profile was significantly worse than that of younger patients (Table 2). Old subjects were frequently admitted with non-ST-elevation ACS, AHF, or bradyarrhythmias (Figure 1(b)) [12]. Elderly patients with ACS had a longer length of stay [4 days, interquartile range (IQR): 3–6 versus 3 days, IQR: 2–5; *p* < 0.0001] and guideline-recommended care was applied less often than their younger counterparts. At multivariable analysis, elderly patients were less likely to receive reperfusion [odds ratio (OR): 0.53, 95% confidence interval (CI): 0.42–0.67] for ST-elevation ACS, or early coronary angiography (OR: 0.45, 95% CI: 0.37–0.56) for non-ST-elevation ACS. Furthermore, patients with ≥2 chronic comorbidities were less likely to receive reperfusion (OR: 0.72, 95% CI: 0.55–0.94; *p* = 0.01). Besides, unadjusted in-ICCU total mortality was higher for elderly patients with ST-elevation (11.8% elderly versus 1.8% younger patients; *p* < 0.0001) or non-ST-elevation (3.9% elderly versus 0.6% younger patients; *p* < 0.0001) ACS [12]. Thus, these data show that the number of elderly patients admitted to the ICCU is substantial. These old subjects are at high risk, often undertreated, and have a worse prognosis. Interestingly, a large French study reported similar results [15]. Thus, in the near future, ICCU standards should consider the complex effects of aging (comorbidities, frailty) on the referring population.

## 5. Acute Heart Failure as an Outline for Modern ICCU

AHF is common and its prevalence is expected to rise in the near future due to aging and chronicization of ACS. In fact, the BLITZ-3 Registry demonstrated that AHF is the most common admission diagnosis after ACS, and among elderly patients AHF prevalence rises significantly [11]. Similarly, the in-ICCU death rate of AHF patients is evenly high as well. On the other hand, while ST-elevation ACS, non-ST-elevation ACS, and arrhythmias have clearly defined targets of care, AHF still appears like a Cinderella disease despite its frequency and ominous nature. This could depend on its complexity and heterogeneity, which challenges the identification of effective standards of care. Furthermore, in the past, cardiologists working in ICCUs have often neglected patients with AHF when this syndrome happened outside the ACS setting. This habit is no more sustainable since it has been clearly demonstrated that AHF patients managed by cardiologists fare better [20, 21]. In fact, competence in echocardiography and other technical skills (noninvasive ventilation, temporary pacing, central venous access, right heart catheterization, etc.) may be useful in the management of these patients. In addition, as opposed to ACS that are most often a “one-shot” accident with a straightforward follow-up, AHF is the acute expression of a chronic pathology that requires a dynamic and specific follow-up after the acute phase. Thus, AHF makes a strong case to justify a specific unit managed by cardiologists or the upgrade of current ICCU standards to include not only optimal coronary care but also high-level heart failure management.

## 6. Appropriateness of Admission and Care

**Figure 3:**
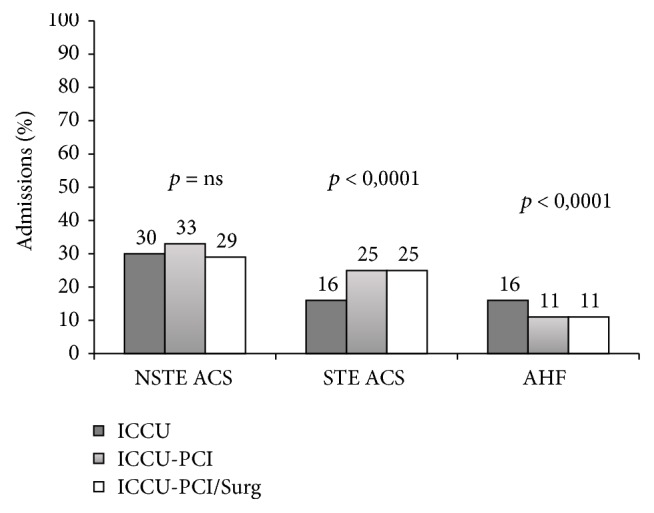
Effects of ICCUs' facilities on epidemiology of admissions. Modified from the BLITZ-3 study, Oltrona Visconti et al. [13].* Legend*. AHF: acute heart failure; ICCU-PCI: Intensive Cardiac Care Units with percutaneous coronary intervention facilities; ICCU-PCI/Surg: Intensive Cardiac Care Units with percutaneous or surgical interventional facilities. Others as Figure 1.

**Figure 4:**
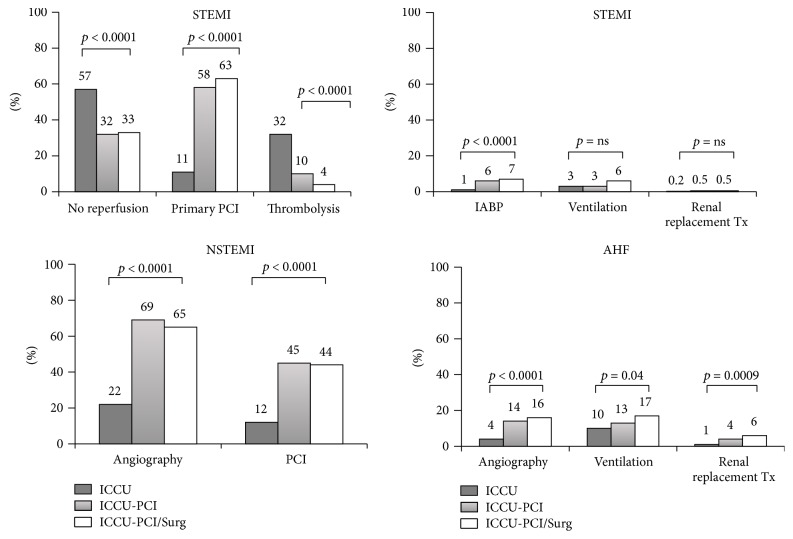
Effects of ICCUs' facilities on diseases management and resource utilization. Modified from the BLITZ-3 study, Oltrona Visconti et al. [13]. Legend as Figures 1 and 3.

Italian surveys' data show that patients cared for in ICCUs are quite heterogenic, ranging from ACS or AHF to cases with non-ACS, non-AHF diseases. This highlights the risk of important clinical and organizational challenges [9, 10]. In fact, we know that patients presenting with acute cardiac conditions managed in specialized cardiac wards have better outcomes. Unfortunately, this is not often the case. An administrative study conducted by the Health Care System of the Lazio Region in Italy, on 9127 patients with acute myocardial infarction hospitalized in Rome from 1997 to 2005, observed that only 54% of these patients were admitted to ICCUs [22]. Of note, younger males, with less severe conditions, fewer comorbidities, and better socioeconomic status, were more frequently admitted to ICCUs. Most importantly, the advantages of primary PCI in ST-elevation ACS, invasive ventilation in advanced respiratory failure, or circulatory support in cardiogenic shock—just to mention the most relevant examples—demonstrate that the availability of these resources could influence ICCU care and outcomes. In the BLITZ-3 survey, the participating ICCUs were classified into three types according to their surgical and interventional facilities: 19% had both interventional facilities and heart surgery (Level 3), 32% had interventional facilities without heart surgery on-site (Level 2), and 49% had neither (Level 1, standard ICCU). Hospital admissions for ST-elevation ACS occurred more frequently in Level 2 or 3 ICCUs (*p* < 0.0001), whereas admission for AHF mostly occurred in Level 1 ICCUs (*p* < 0.0001) (Figure 3). The number of patients not undergoing reperfusion (*p* < 0.0001) or treated with thrombolytic therapy (*p* < 0.0001) was higher in Level 1 ICCUs. Similarly, patients hospitalized for non-ST-elevation ACS underwent coronary angiography (*p* < 0.0001) and PCI more frequently in Level 2 or 3 ICCUs (*p* < 0.0001) (Figure 4). Interestingly, interventional capabilities of ICCUs were the strongest predictor of reperfusion [OR: 2.63, 95% CI: 2.08–3.32, *p* < 0.0001] in ST-elevation ACS and coronary angiography [OR: 8.57, 95% IC: 6.93–10.6, *p* < 0.0001] in non-ST-elevation ACS. Prevalence of low-risk patients was higher in Level 1 ICCUs (*p* < 0.05), while Level 3 ICCUs admitted higher risk cases (*p* < 0.05) [9].

Thus, resources availability preselects patients and impacts acute cardiac care. This could negatively affect the universal quality of care, and new strategies, like networking and transferring according to clinical condition, should be pursued to overcome this problem.

## 7. Effects of Hospital Networks for ST-Elevation ACS on ICCUs' Activity

**Figure 5:**
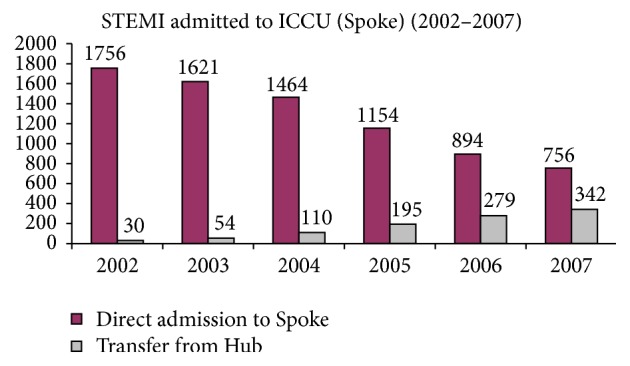
Effects of STEMI network implementation in Emilia-Romagna on ICCUs' activities. The reduction of direct admission to Spoke (Level 1) ICCU is not compensated by the transfer back from the Hub (Level 2 or 3) ICCU of patients initially triaged by EMS directly to the interventional center for reperfusion. Modified from Pavesi et al. [10].* Legend*. STEMI: ST-elevation myocardial infarction.

Resources availability is the strongest driver of ICCU attitudes and could influence admissions to a particular ICCU, levels of care, and outcomes [13] (Figure 4). Interestingly, when between 2002 and 2007 the effects of STEMI networks implementation in the Italian region Emilia-Romagna were assessed, a substantial decline (−14%) of admissions to Level 1 ICCUs was observed (Table 3). These changes in the epidemiology of Level 1 ICCU were largely due to a 57% decrease of the ST-elevation population, not offset by a 20% increase in admissions for non-ST-elevation ACS or by the number of patients transferred back from Level 2 or 3 ICCUs after reperfusion or stabilization (Figure 5). This evidence may be unfavorable to the survival of non-PCI-capable ICCU. However, the reduced number of admissions due to STEMI networking could be compensated by an increase of patients with AHF, or other acute cardiac illnesses (type 2 AMI, etc.) [10]. In fact, these subjects could be shifted from medical wards, where they are often cared for, to Level 1 ICCUs, and we can take advantage of their cardiological competences. Interestingly, networking is always a very effective model of working that could be extended to other non-ACS critical cardiac conditions. In fact, the BLITZ-3 survey observed that a small but consistent number of patients admitted to ICCUs with non-ST-elevation ACS, AHF, or other acute non-ACS, non-AHF cardiac diseases were transferred in from other hospitals. Thus, these subjects were captured from the network although they were not affected by an ST-elevation ACS [9].

In summary, networking is a system of work that improves efficiency. However, it could have dramatic effects on epidemiology and case load of the different ICCUs. In fact, it increases the population of ST-elevation ACS or other complex cases of higher-level ICCU, while it could reduce the activity of peripheral, noninterventional ICCUs. These Level 1 units should compensate this change focusing on non-ACS critical cases that could take advantage of many cardiological competences.

## 8. Clinical Competences of Cardiologists Working in ICCUs

According to the previous findings, it is not surprising that physicians in charge of contemporary ICCUs need to expand their skills. Today, they should be able to recognize and treat a wide variety of acute cardiac conditions and different comorbidities [17]. In addition, they should be familiar with all the diagnostic and therapeutic options available in a modern ICCU. Furthermore, cardiologists in charge need to acquire soft skills, like communication, team working, management, empowerment, and several others [23]. Moreover, contemporary ICCUs are often the core of an integrated acute cardiac care network. In this model, the ICCU of a referring center (Hub) plays a central role in keeping continuous and tight relations with the other peripheral hospitals (Spoke) that have a prominent and unique role in the selection and early treatment of acute cardiac patients and their follow-up. Thus, this evolution of acute cardiac care looks for skilled doctors with a strong attitude to team working and highly specialized ICCU. Consequently, specific training programs on intensive cardiac care for cardiologists working in ICCUs are clearly warranted [24, 25]. Few years ago, these data from the Italian ICCUs surveys stimulated the ANMCO to promote advanced training programs on intensive cardiac care [23]. These projects aimed to improve the quality of care of Italian ICCUs through an update of knowledge and skills of cardiologists in charge.

## 9. Conclusions

The Italian BLITZ-3 survey and other regional experiences have provided unique observations on the evolution of acute cardiac care and ICCUs themselves as well. Although ACS still remains the most frequent admission diagnosis, numbers of AHF cases are substantial. Interestingly, age, resources availability, and networking have a strong influence on ICCUs' activity. In fact, while patients with ACS concentrate in Level 2 or 3 ICCUs with interventional capabilities, older patients with AHF or non-ACS, non-AHF cardiac diseases prevail in peripheral Level 1 ICCUs. Therefore, all these changes challenge current competences and organization of acute cardiac care and promote rapid evolution of ICCUs' organization and competences of cardiologists in charge.

## Figures and Tables

**Table 1:** Clinical characteristics of the BLITZ-3 Registry population. Data are shown for the general population, for patients with ST-elevation acute coronary syndromes (ACS), non-ST-elevation ACS, acute heart failure (AHF), or other acute non-ACS, non-AHF cardiac diseases [9].

	General population (*N* = 6986)	ST-elevation ACS (*N* = 1492)	Non-ST-elevation ACS (*N* = 2144)	Acute heart failure (*N* = 966)	Other non-ACS, non-AHF cardiac diseases (*N* = 2384)
Age, yrs, median (IQR)	72 (61–80)	68 (58–77)	71 (62–79)	76 (67–82)	73 (62–80)
Female gender, %	36	30	33	42	42
Previous relevant cardiac or noncardiac comorbidities, %	70	51	72	94	70
Admission to the emergency room, %	63	62	63	62	64
Direct referral to ICCU by EMS, %	4	8	2	4	3
Transthoracic echocardiography, %	78	84	82	79	72
Coronary angiography, %	35	65	50	10	13
Any PCI, %	24	59	32	1	5
Noninvasive or invasive ventilation, %	4	4	2	14	2
Pulmonary catheter, %	0.5	0.6	0.2	1	0.6
IABP, %	1	5	0.9	0.5	0.5
Ultrafiltration, %	1	0.4	0.7	3	0.7
Temporary pacing, %	4	2	0.6	0.9	8
Inotropes, intravenous, %	8	10	4	22	7
Diuretics, intravenous, %	47	35	39	93	43
Insulin, subcutaneous or intravenous, %	19	18	21	28	13
Transfusions, %	4	3	4	7	3
Major ventricular arrhythmias, %	4	6	2	4	3
High-grade AV Block, %	2	3	1	0.8	3
Fatal or nonfatal stroke, %	0.5	0.6	0.6	0.8	0.3
Heart failure or worsening, %	9	12	10	12	5
Shock or Killip IV, %	6	6	2	20	2
Cardiac arrest, %	3	5	2	4	2
Sepsis, %	0.8	0.2	0.6	2	0.7
Acute renal dysfunction, %	11	13	11	18	8
Length of stay in ICCU, median (IQR)	4 (2–5)	4 (3–5)	4 (3–6)	4 (3–6)	3 (2–4)
In-ICCU crude global mortality	3.3	5.1	2	5.4	2.6

IQR: interquartile range; ICCU: Intensive Cardiac Care Unit; EMS: Emergency Medical Services; PCI: percutaneous coronary intervention; IABP: intra-aortic balloon pump; AV: atrioventricular.

**Table 2:** Main clinical characteristics of the BLITZ-3 Registry population according to age subgroups [9].

	Age < 75 (*N* = 4014)	Age ≥ 75 (*N* = 2972)	*p*
Female gender, %	27	49	<0.0001
Diabetes, %	23	27	<0.0001
Previous myocardial infarction, %	22	27	<0.0001
Previous stroke or PVD, %	10	20	<0.0001
Atrial fibrillation, %	8	20	<0.0001
Neoplasm, %	4	8	<0.0001
No comorbidities, %	39	18	<0.0001
Creatinine > 2 mg/dl on admission, %	5	12	<0.0001
Hemoglobin < 10 gr/dl on admission, %	5	10	<0.0001
Renal failure in ICCU, %	9	15	<0.0001
Heart failure in ICCU, %	6	12	<0.0001
Shock or Killip IV in ICCU, %	3,5	8,1	<0.0001
High-grade AV block in ICCU, %	1,8	2,9	0.003
Cardiac arrest in ICCU, %	1,4	5	<0.0001
Length of stay in ICCU, median (IQR)	3 (2–5)	4 (3–6)	<0.0001
In-ICCU crude global mortality	1.35	6.03	<0.0001

IQR: interquartile range; AV: atrioventricular; ICCU: Intensive Cardiac Care Unit; PVD: peripheral vascular disease.

**Table 3:** Effects of STEMI network implementation on ICCUs activities. Data from the Italian Emilia-Romagna ICCUs Network. Modified from Pavesi et al. [10].

STEMI	ICCU with interventional capabilities (Hub)			ICCU without interventional capabilities (Spoke)		
	2002	2007	*p*	2002	2007	*p*
Patients, number	2450	2873	<0.0001	1756	756	<0.0001
Male gender, %	66.8	68.5	0.44	67.4	63.5	0.003
Age (median), yrs (IQR)	70 (59–79)	68 (58–78)	0.07	71 (59–79)	73 (61–82)	0.0002
>2 comorbidities, %	12.6	11.7	0.02	13	20.5	<0.0001
PCI < 24 h, %	24.1	76.5	<0.0001	6.1	35.1	<0.0001
In-hospital crude mortality, %	14.2	11.3	0.0002	11.9	10.4	0.87
One-year crude mortality, %	22.2	18.3	<0.0001	20.1	20.5	0.08

IQR: interquartile range; ICCU: Intensive Cardiac Care Unit; PCI: percutaneous coronary intervention.

## References

[1] Julian D. (1961). Treatment of cardiac arrest in acute myocardial ischæmia and infarction. *The Lancet*.

[2] Morrow D. A., Fang J. C., Fintel D. J. (2012). Evolution of critical care cardiology: transformation of the cardiovascular intensive care unit and the emerging need for new medical staffing and training models: a scientific statement from the American Heart Association. *Circulation*.

[3] Katz J. N., Turer A. T., Becker R. C. (2007). Cardiology and the critical care crisis. A perspective. *Journal of the American College of Cardiology*.

[4] Gardini E., Caravita L., Ottani F., Ferrini D., Galvani M. (2007). Coronary care units: who to admit and how long. *Giornale Italiano Di Cardiologia*.

[5] Lettino M., Vrints C. (2013). Acute cardiovascular care IV.. *European Heart Journal*.

[6] Acute Cardiac Care Association - European Society of Cardiology ACCA white book 2016- first edition. https://www.escardio.org/static_file/Escardio/Subspecialty/ACCA/Documents/ACCA%20WB%20prefinal%20ED%20DC%20Final.pdf.

[7] O’malley R. G., Olenchock B., Bohula-May E. (2013). Organization and staffing practices in US cardiac intensive care units: a survey on behalf of the American Heart Association Writing Group on the Evolution of Critical Care Cardiology. *European Heart Journal: Acute Cardiovascular Care*.

[8] Hasin Y., Danchin N., Filippatos G. S. (2005). Recommendations for the structure, organization, and operation of intensive cardiac care units. *European Heart Journal*.

[9] Casella G., Cassin M., Chiarella F. (2010). Epidemiology and patterns of care of patients admitted to Italian Intensive Cardiac Care units: the BLITZ-3 registry. *Journal of Cardiovascular Medicine*.

[10] Pavesi P. C., Nobilio L., De Palma R., Casella G., Di Pasquale G., Grilli R. (2011). The evolution of intensive cardiac care units and the effects of interhospital networks for reperfusion implementation. Analysis of the Emilia-Romagna regional data, 2002-2007. *Giornale Italiano Di Cardiologia*.

[11] Chinaglia A., Casella G., Scorcu G. (2012). on behalf of the Blitz-3 Investigators. Heart failure in Italian intensive cardiac care units: data from the BLITZ-3 Registry. *Giornale Italiano Di Cardiologia*.

[12] Casella G., Scorcu G., Cassin M. (2012). Elderly patients with acute coronary syndromes admitted to Italian intensive cardiac care units: a Blitz-3 Registry sub-analysis. *Journal of Cardiovascular Medicine*.

[13] Oltrona Visconti L., Scorcu G., Cassin M. (2011). Distribution and appropriateness of hospital admissions, resources utilization in the Italian intensive cardiac care units. The BLITZ-3 study. *Giornale Italiano Di Cardiologia*.

[14] Valente S., Lazzeri C., Sori A., Giglioli C., Bernardo P., Gensini G. F. (2007). The recent evolution of coronary care units into intensive cardiac care units: the experience of a tertiary center in Florence. *Journal of Cardiovascular Medicine*.

[15] Roubille F., Mercier G., Delmas C. (2017). Description of acute cardiac care in 2014: a French nation-wide database on 277,845 admissions in 270 ICCUs. *International Journal of Cardiology*.

[16] Holland E. M., Moss T. J. (2017). Acute noncardiovascular illness in the cardiac intensive care unit. *Journal of the American College of Cardiology*.

[17] Dudzinski D. M., Januzzi J. L. (2017). The evolving medical complexity of the modern cardiac intensive care unit. *Journal of the American College of Cardiology*.

[18] van Diepen S., Bakal J. A., Lin M., Kaul P., McAlister F. A., Ezekowitz J. A. (2015). Variation in critical care unit admission rates and outcomes for patients with acute coronary syndromes or heart failure among high- and low-volume cardiac hospitals. *Journal of the American Heart Association*.

[19] Alonso J. J., Sanz G., Guindo J., García-Moll X., Bardají A., Bueno H. (2007). Intermediate coronary care units: rationale, infrastructure, equipment, and referral criteria. *Revista Española de Cardiología*.

[20] Adams K. F., Fonarow G. C., Emerman C. L. (2005). Characteristics and outcomes of patients hospitalized for heart failure in the United States: rationale, design, and preliminary observations from the first 100,000 cases in the Acute Decompensated Heart Failure National Registry (ADHERE). *American Heart Journal *.

[21] Nicol E. D., Fittall B., Roughton M., Cleland J. G. F., Dargie H., Cowie M. R. (2008). NHS heart failure survey: a survey of acute heart failure admissions in England, Wales and Northern Ireland. *Heart*.

[22] Saitto C., Ancona C., Fusco D., Arcà M., Perucci C. A. (2004). Outcome of patients with cardiac diseases admitted to coronary care units a report from Lazio, Italy. *Medical Care*.

[23] Fradella G., De Luca L., Tubaro M. (2010). Clinical competence in intensive cardiac care units: from practical needs to training programs. *Giornale Italiano di Cardiologia*.

[24] O'Gara P. T., Adams J. E., Drazner M. H. (2015). COCATS 4 task force 13: training in critical care cardiology. *Journal of the American College of Cardiology*.

[25] Casella G., Di Pasquale G. (2007). Clinical competence of the cardiologists working in coronary care units. *Giornale Italiano Di Cardiologia*.

